# Minimum Inhibitory Concentration and Antibiotic Sensitivity Patterns in Pediatric Patients With Urinary Tract Infections

**DOI:** 10.7759/cureus.80831

**Published:** 2025-03-19

**Authors:** Abhishek Ranjan, Priya S Lakra, Shyam S Sahu, Ankita, Kavita Tirkey, Vidisa Bose, Hirendra Birua

**Affiliations:** 1 Department of Pediatric Surgery, Rajendra Institute of Medical Sciences, Ranchi, IND

**Keywords:** antibiotics, antibiotic susceptibility testing, bacterial pathogens, pediatrics, urinary tract infections

## Abstract

Background

Clinical professionals find it challenging to set uniform standards for the treatment of urinary tract infections (UTIs) because of differences in susceptibility. This study has been conducted to assess antibiotic sensitivity patterns and bacterial profiles in pediatric patients with UTI and aid in the empirical management of patients exhibiting UTI symptoms.

Materials and methods

This is an observational prospective study conducted at the Department of Pediatric Surgery at Rajendra Institute of Medical Sciences (RIMS), Ranchi, Jharkhand, India. Overall, 384 patients were enrolled in the study. Ethical approval was obtained from the Institutional Ethics Committee (IEC), RIMS, Ranchi, Jharkhand, India, under letter number 308 dated April 4, 2024.

Results

There was no significant difference in age groups between male and female participants. Various bacterial pathogens were detected in pediatric UTIs, including *Escherichia coli*, *Proteus* species, *Klebsiella* species, *Enterobacter* species, *Serratia* species, *Citrobacter* species, and *Pseudomonas* species. *Escherichia coli* was more sensitive to ertapenem (135 (93.1%)) and meropenem (130 (89.6%)), followed by amikacin (118 (81.3%)), nitrofurantoin (115 (79.3%)), and imipenem (111 (76.5%)).

Conclusion

Different antibiotics have shown different levels of sensitivity toward various strains of bacterial pathogens causing UTIs. It is recommended that antibiotics as a medication be suggested on the basis of antibiotic sensitivity patterns in different cases of UTIs. Also, regional data may be effective in the selection of suitable drugs.

## Introduction

Urinary tract infections (UTIs) tend to be more common in both outpatient and inpatient settings in healthcare facilities and communities [1]. Due to their ambiguous and mild symptoms, young children frequently go undiagnosed with UTIs. Chronic renal failure and hypertension can result from renal scarring in young children's growing renal cortex. A urine culture report should be used to characterize and document a clinically suspected UTI case. This aids in determining the best antimicrobial agent, the length of treatment, the necessity of chemoprophylaxis, and the proper evaluation of radio/nuclear imaging [2].

Fever and even sepsis-like symptoms in infants and children that are below two years of age are some major indicators of UTI. However, signs were different in children who were older than two years. Those children may exhibit pain during voiding, urine discoloration, or a poor stream [3]. Along with systemic involvement inclusive of fever, chills, flank pain, and tenderness in the costovertebral angle, children with pyelonephritis also had urine symptoms [4]. For healthcare facilities, nosocomial infections pose serious financial and public health challenges [5].

With advancements in diagnostic technology, improvements in care, and novel medications, UTIs should be precisely checked, as an incorrect diagnosis can lead to poor treatment and prognosis [6]. Additionally, etiology, antimicrobial susceptibility patterns, and clinical presentation differ by area [7]. *Escherichia coli* accounts for 80%-90% of the most frequent pathogens isolated from urine cultures [8]. *Proteus*, *Citrobacter*, *Enterobacter*, and *Serratia* species are among the other bacteria that were formerly uncommonly isolated but are now becoming more prevalent [9].

It has been considered that extended-spectrum β-lactamase (ESBL) enzymes, which are produced by* E. coli*, offer resistance to medications such as monobactams, extended-spectrum cephalosporins, and penicillin. Because of improper antibiotic regimens, these ESBL-producing bacteria are linked to higher rates of morbidity and mortality [10]. There are several factors to consider while choosing antibiotics. Antimicrobial susceptibility is one of the most important aspects to take into account because it might change depending on the microbe's habitat and time. Thus, comprehensive and regular testing for antibiotic susceptibility is required [11]. Trimethoprim-sulfamethoxazole (TMP-SMX) and β-lactam antibiotics continue to be the first-choice therapy for pediatric UTIs despite rising antibiotic resistance [9].

The purpose of this research is to evaluate the bacterial profile and patterns of antibiotic sensitivity in pediatric patients with UTIs and to aid in the clinical management of patients exhibiting UTI symptoms.

## Materials and methods

Study design

This is an observational prospective study conducted at the Department of Pediatric Surgery at Rajendra Institute of Medical Sciences (RIMS), Ranchi, Jharkhand, India. The study was conducted for six months (from April 2024 to September 2024).

Study population

A total of 384 patients were enrolled in the study. Initially, 552 patients were assessed, among which only 384 patients had positive cultures. The study included all pediatric inpatients who were suspected of having UTI and presented at the Department of Pediatric Surgery, RIMS, Ranchi, Jharkhand, India. Patients who had a history of fever (greater than 38°C) or UTI symptoms (dysuria, hesitation, frequency, urgency, low-volume voids, or lower abdominal discomfort) were eligible to participate in the study. The growth of a single pathogen with more than 10 × 5 colony-forming units/mL (CFU/mL) in a properly collected urine specimen (suprapubic aspiration, transurethral catheterization, or midstream urine) from children with fever or other urinary symptoms was considered a urinary tract infection in infants and children. The exclusion criteria were patients who left before completing treatment and critically ill patients requiring hospitalization, prior history of urinary infections (recorded in a prior urine culture sensitivity report), known urinary malformations (based on prenatal ultrasound and prior medical records), chronic illnesses, or ongoing preventative care or antibiotic pretreatment.

Data collection

Laboratory data such as the presence of various bacterial pathogen specimens in the pediatric population were taken into consideration and hence presented below. The procedures used to process urine samples were culture, gram staining, and urine microscopy. Wet mount microscopy was used to identify white blood cells (WBCs), red blood cells, yeast, and epithelial cells in a well-mixed, uncentrifuged urine sample. The gram stain and biochemical reactions were used to identify the isolates.

Study procedure

For the sample collection procedure, it was suggested to collect midstream urine in older children. However, for children who were below two years of age, the procedure that was followed was transurethral bladder catheterization or suprapubic aspiration to obtain urine samples. Urine culture was then performed on either nutrient agar or MacConkey agar with a calibrated loop. Minimum inhibitory concentration (MIC) was determined for urine culture. The antibiotic sensitivity test was conducted on Mueller-Hinton agar. The method that was preferred was the Kirby-Bauer disc diffusion method. Double disc synergy tests were also done.

Bacterial isolates were detected based on the results of gram stain, culture, and WBC counts. All detected bacterial isolates were further examined for antibiotic sensitivity. The method that was used for the disc diffusion technique was performed in line with the guidelines that were provided by the Clinical Laboratory Standard Institute (CLSI) [12]. Our study included the following antibiotics: ceftazidime (30 μg), cefoxitin (30 μg), piperacillin-tazobactam (36 μg), imipenem (10 μg), trimethoprim-sulfamethoxazole (1.25/23.75 μg), meropenem (10 μg), ertapenem (10 μg), levofloxacin (5 μg), cefepime (30 μg), cefotaxime (30 μg), ceftriaxone (30 μg), ciprofloxacin (5 μg), ampicillin (10 μg), amoxicillin-clavulanate (20/10 μg), cephalothin (30 μg), cefuroxime (30 μg), gentamicin (10 μg), amikacin (30 μg), tigecycline (15 μg), nitrofurantoin (300 μg), and colistin (10 μg).

Statistical analysis

Data were entered and templates generated in MS Excel version 13 (Microsoft Corp., Redmond, WA). Data were analyzed using SPSS software version 24 (IBM Corp., Armonk, NY). Data were presented as numbers (%) or simply as percentages. The unpaired t-test was used to compare variables of different groups. A p-value below 0.05 was considered to be significant.

Ethical clearance

Ethical approval was obtained from the Institutional Ethics Committee (IEC) of RIMS, Ranchi, Jharkhand, India, under letter number 308 on April 4, 2024.

## Results

Table 1 shows the age group distribution of the pediatric patients. Most of the participants were in the age group of 5-8 years (110 (28.6%)), followed by 8-15 years (96 (25%)), 1-2 years (68 (17.7%)), 2-5 years (63 (16.4%)), and 0-12 months (47 (12.2%)). Most of the enrolled participants were female (202 (52.6%)). No significant differences were observed in the age groups between male and female participants.

**Table 1:** Age Group Distribution Data was presented as numbers (%). p-value was considered significant at <0.05. The unpaired t-test was used to obtain the p-value.

Age group	Males (n=182)	Females (n=202)	t-statistics value	p-value
0-12 months	21 (11.5%)	26 (12.8%)	0.39	0.34
1-2 years	31 (17.03%)	37 (18.3%)	0.32	0.37
2-5 years	29 (15.9%)	34 (16.8%)	0.23	0.4
5-8 years	59 (32.4%)	51 (25.2%)	-1.5	0.06
8-15 years	42 (23.07%)	54 (26.7%)	0.8	0.2

Various bacterial pathogens were detected in pediatric UTIs, including *Serratia* species,* Pseudomonas* species, *Enterobacter* species, *Citrobacter* species, *Proteus* species, *Klebsiella* species, and *E. coli*. Table 2 represents bacterial pathogens involved in pediatric urinary tract infections.

**Table 2:** Bacterial Pathogens in Pediatric UTIs Data was presented as numbers (%). UTIs: urinary tract infections

Bacterial pathogens	Values
Escherichia coli	145 (37.7%)
*Klebsiella* species	88 (22.9%)
*Proteus* species	3 (0.7%)
*Enterobacter* species	18 (4.6%)
*Citrobacter* species	32 (8.3%)
*Serratia* species	29 (7.5%)
*Pseudomonas* species	69 (17.9%)

Table 3 depicts the minimum inhibitory concentration for and antibiotic sensitivity patterns of different pathogens involved. *Escherichia coli* was more sensitive to ertapenem (135 (93.1%)) and meropenem (130 (89.6%)), followed by amikacin (118 (81.3%)), nitrofurantoin (115 (79.3%)), and imipenem (111 (76.5%)). *Klebsiella* species were more sensitive to ertapenem (69 (78.4%)) and meropenem (65 (73.8%)), followed by imipenem and levofloxacin. Most of the highest activity has been shown by imipenem, ertapenem, and meropenem among all the enlisted antibiotics in different species.

**Table 3:** Minimum Inhibitory Concentration and Antibiotic Sensitivity Data were presented as numbers (%). NT: not tested

Antibiotics	*Escherichia coli* (n=145)	*Klebsiella* species (n=88)	*Proteus* species (n=3)	*Enterobacter* species (n=18)	*Citrobacter* species (n=32)	*Serratia* species (n=29)	*Pseudomonas* species (n=69)
Ampicillin	55 (37.9%)	72 (81.8%)	1 (33.3%)	0 (0%)	0 (0%)	0 (0%)	NT
Amoxicillin-clavulanate	89 (61.3%)	37 (42%)	1 (33.3%)	0 (0%)	18 (56.2%)	0 (0%)	NT
Cephalothin	46 (31.2%)	35 (39.7%)	1 (33.3%)	0 (0%)	11 (34.3%)	0 (0%)	NT
Cefuroxime	62 (42.7%)	26 (29.5%)	3 (100%)	13 (72.2%)	20 (62.5%)	0 (0%)	NT
Ceftazidime	57 (39.3%)	34 (38.6%)	2 (66.6%)	16 (88.8%)	16 (50%)	22 (75.8%)	32 (46.3%)
Cefoxitin	43 (29.6%)	45 (51.1%)	2 (66.6%)	0 (0%)	26 (81.2%)	0 (0%)	NT
Cefepime	51 (35.1%)	28 (31.8%)	3 (100%)	17 (94.4%)	22 (68.7%)	17 (58.6%)	39 (56.5%)
Cefotaxime	76 (52.4%)	45 (51.1%)	2 (66.6%)	13 (72.2%)	23 (71.8%)	19 (65.5%)	NT
Ceftriaxone	93 (64.1%)	27 (30.6%)	3 (100%)	12 (66.6%)	20 (62.5%)	26 (89.6%)	NT
Ciprofloxacin	43 (29.6%)	52 (59%)	2 (66.6%)	13 (72.2%)	16 (50%)	21 (72.4%)	39 (56.5%)
Gentamicin	28 (19.3%)	31 (35.2%)	2 (66.6%)	17 (94.4%)	24 (75%)	27 (93/1%)	46 (66.6%)
Amikacin	118 (81.3%)	52 (59%)	1 (33.3%)	13 (72.2%)	29 (90.6%)	25 (86.2%)	33 (47.8%)
Trimethoprim-sulfamethoxazole	57 (39.3%)	26 (29.5%)	3 (100%)	12 (66.6%)	23 (71.8%)	17 (58.6%)	NT
Piperacillin-tazobactam	47 (32.4%)	27 (30.6%)	2 (66.6%)	13 (72.2%)	22 (68.7%)	25 (86.2%)	49 (71%)
Imipenem	111 (76.5%)	63 (71.5%)	3 (100%)	17 (94.4%)	20 (62.5%)	23 (79.3%)	39 (56.5%)
Meropenem	130 (89.6%)	65 (73.8%)	2 (66.6%)	14 (77.7%)	31 (96.8%)	14 (48.2%)	27 (39.1%)
Ertapenem	135 (93.1%)	69 (78.4%)	2 (66.6%)	15 (83.3%)	22 (68.7%)	14 (48.2%)	NT
Levofloxacin	104 (71.7%)	59 (67%)	2 (66.6%)	14 (77.7%)	23 (71.8%)	26 (89.6%)	56 (81.1%)
Nitrofurantoin	115 (79.3%)	43 (48.8%)	1 (33.3%)	12 (66.6%)	16 (50%)	NT	NT

## Discussion

The study presented antibiotic sensitivity patterns and bacterial profiles in pediatric patients with urinary tract infections. The study involved 384 pediatric participants. Most of the affected children with UTI were of age group 5-8 years, followed by 8-15 years, 1-2 years, 2-5 years, and 0-12 months.

In our study, the percentages of female participants were higher than male participants who had confirmed UTI. Two other research conducted by Mirsoleymani et al. [13] and Farajnia et al. [14] found that the ratio of male participants to female participants was 1:4 and 1:2, respectively. A recent research conducted by Shaaban et al. also revealed that the percentage of confirmed UTIs was 40.76% and 43.54% among male and female participants, respectively, who were suspected of having a UTI [15]. Other than this, no statistical differences were observed between male and female participants in age groups.

It has been observed that *E. coli* was the prominent cause of UTI in children in our study. Similar results have been reported by Altamimi et al. in a study conducted in 2023 that *E. coli* was the major cause of UTI [16]. The same results that *E. coli* is the main pathogen causing UTI are observed with rates of 58%, 64.2%, and 75.7% in the studies by Mamishi et al. [8], Gunduz et al. [11], and Odoki et al. [17], respectively. However, according to the study by Heidari et al.,* P. aeruginosa *is the most frequent infection that causes UTIs [18].

The second most common pathogen that causes UTIs is *Klebsiella* species, followed by *Pseudomonas *and *Citrobacter* species. In a similar study, the second most common bacteria that was found to cause UTI was* Klebsiella pneumoniae*, with a rate of 3% among a pediatric sample of 361 children [19]. An Iran-based study demonstrated more evidence for this, showing that 36.2% of UTIs in children under five are caused by *Klebsiella* species [13]. However, according to Turkish research, *Proteus* species tended to be the second most prevalent organism in the pediatric age group to cause UTIs, with *Klebsiella* coming in after [20].

It was observed that *E. coli* was more delicate toward ertapenem and meropenem, followed by amikacin, nitrofurantoin, and imipenem. Also, *E. coli* is highly resistant to cefazolin, followed by another drug ampicillin and ceftazidime [15]. Among children with confirmed UTI, resistance was highest in the ampicillin group [13,18,20,21]. Furthermore, it was discovered that *E. coli* had the highest nitrofurantoin sensitivity rate of 98.96%, with a p-value of less than 0.01, followed by amikacin with a rate of 98.43%, with a p-value of less than 0.01. This was consistent with recent research showing that *E. coli* had a high sensitivity to nitrofurantoin, ranging from 90.5% to >99% [13,18,20-23]. Various other strains of species were more sensitive to different antibiotics including ampicillin, cefuroxime, amoxicillin-clavulanate, and ceftazidime. Further treatments on the basis of antibiotic sensitivity testing were provided to the participants for better results.

One of the study's limitations was that it only looked at patients who were hospitalized and kept them under observation until the time of sample collection, without looking into any illnesses that could have arisen after the patients were released. Another limitation was the sample size, which might be due to convenience sampling and the duration of the study. The study only collected data relevant to antibiotic susceptibility testing, and data regarding routine consultations or emergencies was not collected or assessed.

## Conclusions

The study concluded that most of the pediatric population with UTIs were between five and eight years of age. Also, more female participants tended to have UTIs as compared to male participants. *Escherichia coli* was the prominent cause of UTIs in children, followed by *Klebsiella* species. Different antibiotics were sensitive to various strains causing urinary tract infections. It is recommended that antibiotics as a medication be suggested based on antibiotic sensitivity patterns in different cases with UTIs. Also, regional data may be effective in the selection of suitable drugs.
